# Efficacy and safety of dapagliflozin in patients receiving dialysis: a post-hoc analysis of the DAPA-CKD trial

**DOI:** 10.1093/ckj/sfag170

**Published:** 2026-05-25

**Authors:** Wisanne M Bakker, Niels Jongs, Glenn M Chertow, Ricardo Correa-Rotter, Peter Rossing, Robert D Toto, David C Wheeler, Hiddo J L Heerspink, Ron T Gansevoort

**Affiliations:** Department of Nephrology, University Medical Center Groningen, University of Groningen, Groningen, the Netherlands; Department of Clinical Pharmacy and Pharmacology, University of Groningen, University Medical Center Groningen, Groningen, Netherlands; Departments of Medicine and Epidemiology and Population Health, Stanford University School of Medicine, Stanford, USA; The National Medical Science and Nutrition Institute Salvador Zubiran, Mexico City, Mexico; Steno Diabetes Center Copenhagen, Gentofte, Denmark; Department of Clinical Medicine, University of Copenhagen, Copenhagen, Denmark; Department of Internal Medicine, UT Southwestern Medical Center, Dallas, TX, USA; Department of Renal Medicine, University College London, London, UK; Department of Clinical Pharmacy and Pharmacology, University of Groningen, University Medical Center Groningen, Groningen, Netherlands; The George Institute for Global Health, University New South Wales, Sydney, Australia; Department of Nephrology, University Medical Center Groningen, University of Groningen, Groningen, the Netherlands

To the Editor,

Patients receiving maintenance dialysis are known to be at high risk for cardiovascular disease and mortality, but therapeutic options to reduce these risks are limited. Over the past years, clinical trials showed the beneficial effects of sodium glucose co-transporter 2 (SGLT2) inhibitors on kidney and cardiovascular outcomes as well as mortality in patients with chronic kidney disease (CKD) [1, 2]. It has been assumed that SGLT2 inhibitors may be less effective in patients with advanced CKD owing to the presence of fewer functional nephrons, with fewer proximal tubular SGLT2 transporters available to inhibit. However, recent experimental evidence suggests that SGLT2 inhibitors may improve clinical outcomes also in these patients via effects independent of functional nephron mass [2].

To explore the effects of SGLT2 inhibition among patients receiving maintenance dialysis, we conducted a post-hoc analysis of the DAPA-CKD (Dapagliflozin And Prevention of Adverse Outcomes in Chronic Kidney Disease) trial in the subgroup of participants who initiated dialysis during the trial. The primary endpoint of the DAPA-CKD trial was a composite including incident kidney failure, which, of course, cannot be assessed in patients already receiving dialysis. Thus, in the current analysis, we focused on the effects of dapagliflozin on all-cause mortality and safety. A detailed description of the Methods can be found in the (Supplementary material, Item S1).

Out of the total study population of the DAPA-CKD trial (n = 4304), 167 participants initiated dialysis during the study. Of these patients 68 were originally assigned to dapagliflozin and 99 to placebo at randomization. Characteristics at start of dialysis are provided in Table S1. Treatment with study drug was continued in 28 (41%) participants in the dapagliflozin group and in 36 (36%) participants in the placebo group (Supplementary material, Table S2). The mean eGFR at start of dialysis was 14.5 ± 10.4 ml/min/1.73m2 for the patients in the dapagliflozin group and 13.8 ± 7.3 ml/min/1.73m2 in the placebo group, *P* = 0.63. The mean urinary albumin-to-creatinine ratio (UACR) at dialysis initiation was 3260.8 ± 2463.5 mg/g for the dapagliflozin group versus 4049.6 ± 2538.2 mg/g in the placebo group, *P* = 0.047 (Supplementary material, Table S1).

Because patient characteristics differed between groups at dialysis initiation, the effects of dapagliflozin on mortality thereafter was adjusted for age, sex, HbA1c, eGFR and UACR. Participants in the dapagliflozin group had significantly lower all-cause mortality (adjusted hazard ratio [aHR] 0.47, 95% CI: 0.23–0.98, Figure 1). When the cause of death was investigated, more participants appeared to have died from a non-cardiovascular cause when compared to a cardiovascular cause (25 versus 12, respectively). Non-CV mortality was significantly reduced in the dapagliflozin group (aHR 0.27, 95% CI: 0.10–0.73), whereas there was no significant effect on cardiovascular mortality (aHR 1.33, 95% CI: 0.40–4.40, Figure 1). The adjusted hazard ratios for these efficacy outcomes according to treatment status are also given in the Supplementary material (Supplementary material, Figure S1).

**Figure 1:**
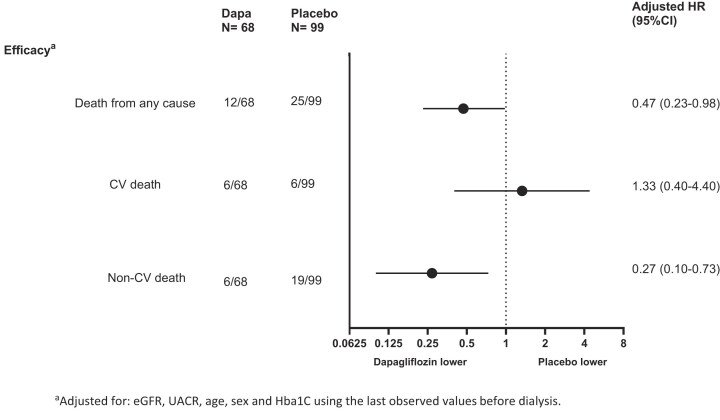
Efficacy after initiation of dialysis.

Our findings regarding safety of SGLT-2 inhibitors in patients on dialysis are in line with previously published studies [3, 4] and add further evidence suggesting that SGLT2 inhibitors can be safely used in patients receiving maintenance dialysis (Supplementary material, Table S3).

Overall, these findings suggest favourable effects for dapagliflozin in dialysis patients on all-cause mortality driven by effects on non-cv mortality. Of course, these data should be interpreted with caution as the number of patients included in this analysis was low, and due to the non-randomized post-hoc design characteristics of subjects at start of dialysis differed between both study groups. Nevertheless, these findings are hypothesis generating and suggest that dapagliflozin may be beneficial and safe in hemodialysis patients.

This hypothesis will be tested in the prospective, randomized Renal Lifecycle trial (NCT 05374291) that will determine the effects of dapagliflozin 10 mg once daily on risks of mortality, heart failure admission and incident kidney failure as well as safety in patients with advanced CKD (eGFR <25 mL/min/1.73m^2^), a kidney transplant and around 600 patients receiving maintenance dialysis [5]. This trial that started in 2022 is expected to be completed in due course. Hopefully, its results will provide the necessary evidence to address the current knowledge gaps in these specific subgroups with advanced CKD.

## Supplementary Material

sfag170_Supplemental_Files

## Data Availability

The data supporting this study may be obtained in accordance with AstraZeneca’s data sharing policy described at https://astrazenecagrouptrials.pharmacm.com/ST/submission/Disclosure.
