# Supplementary material for: Efficacy and safety of dapagliflozin in patients receiving dialysis: a post-hoc analysis of the DAPA-CKD trial
Source: Clin Kidney J. 2026 May 25;19(7):sfag170. doi: 10.1093/ckj/sfag170 (PMC13339949; doi:10.1093/ckj/sfag170)
Supplement: sfag170_Supplemental_Files [file sfag170_supplemental_files.zip › Supplementary material Efficacy and safety of dapagliflozin in patients receiving dialysis.docx]

**Supplementary material**

**Efficacy and safety of dapagliflozin in patients receiving dialysis**

**A post-hoc analysis of the DAPA-CKD trial**

**Contents**

**Item S1.** Methods

**Table S1.** Characteristics of participants at start of dialysis

**Table S2.** Characteristics of participants at start of dialysis according to treatment status.

**Figure S1:** Efficacy after initiation of dialysis according to treatment status

**Table S3.** Safety after initiation of dialysis according to treatment status

**Item S1.** Methods

A detailed description of the trial design was published elsewhere [1].

*Participants*

Patients needed to receive a stable dose of an angiotensin-converting enzyme (ACE) inhibitor or Angiotensin II receptor blocker (ARB) at least 4 weeks prior to screening. In case of documentation of intolerance or inability to take ACE inhibitors or ARBs patients were permitted to participate. Exclusion criteria consisted of type 1 diabetes, polycystic kidney disease, lupus nephritis, or anti-neutrophil cytoplasmic antibodies (ANCA)- associated vasculitis and immunotherapy for a primary or secondary kidney disease within 6 months prior to enrolment.

*Trial procedures*

Once eligibility was proven through screening participants were randomly assigned to dapagliflozin 10 mg once daily or matching placebo (1:1) on top of standard care. Randomization was stratified by UACR (or >1000 mg/g) and diabetes status. This was a double-blind trial were both study personnel and participants were masked to treatment allocation. After randomization follow-up visits took place at two weeks, 2, 4, and 8 months, and hereafter every 4 months. Study visits included assessments of vital signs, blood-and urine sampling and recording of adverse events, study endpoints, drug adherence and concomitant therapies.

*Trial outcomes*

Prespecified endpoints of the DAPA-CKD trial were as follows, the primary composite endpoint was of time to first appearance of a sustained ≥50% decline in eGFR, onset of kidney failure (defined as kidney transplantation, provision of dialysis or an eGFR <15 mL/min/.1.73m2), or death due to a kidney or cardiovascular cause. Secondary outcomes included a kidney composite endpoint (i.e., the primary composite endpoint without cardiovascular death), a cardiovascular composite endpoint (i.e., hospitalization for heart failure or cardiovascular death), and all-cause mortality. Based on pre-specified definitions, events were assessed by an independent event adjudication committee. In this post-hoc analysis we assessed the occurrence of adverse events and mortality in a specific subgroup of participants who initiated dialysis during the trial to gain insight in safety and efficacy in this subgroup.

*Statistical analysis*

Characteristics of the study population at the start of dialysis are presented by treatment group (dapagliflozin versus placebo). Continuous data are presented as mean (SD). We calculated odds ratios (OR) with 95% confidence intervals (95% CI) for the occurrence of adverse events of dapagliflozin 10 mg versus placebo. We performed cox-proportional hazard regression analysis to investigate mortality rates at start of dialysis as baseline. Analyses were censored for event, death and end of follow-up. These analyses were adjusted for baseline eGFR and stratified by baseline UACR, type 2 diabetes status, baseline age and HBa1C as this is a non-randomized population with differences in participant characteristics between the dapagliflozin group and the placebo group at start of dialysis. A p-value <0.05 was regarded as statistically significant. Analysis were performed using R (version 4.5.1, R core team, 2025).

**Table S1: Characteristics of participants at start of dialysis**

|  |  |  |  |
| --- | --- | --- | --- |
|  | **Dapagliflozin**  **(n=68)** | **Placebo**  **(n=99)** | **P-value** |
| **Age** - yr | 57.4 ± 13.7 | 55.1 ± 14.4 | 0.309 |
| **Sex** (female) - no. (%) | 17(25) | 31 (31.3) | 0.477 |
| **Race** – no. (%) |  |  | 0.756 |
| - Asian | 19 (27.9) | 23 (23.2) |  |
| Black or African American | 3 (4.4) | 3 (3) |  |
| White | 37 (54.4) | 55 (55.6) |  |
| Other | 9 (13.2) | 18 (18.2) |  |
| **CV event before dialysis**- no. (%) |  |  |  |
| - No | 40 (58.8) | 61 (61.6) | 0.840 |
| - Yes | 28 (41.2) | 38 (38.4) |  |
| **Reason of kidney failure**- no. (%) |  |  | 0.556 |
| Acute renal failure | 15 (22.1) | 17 (17.2) |  |
| Progression of disease | 53 (77.9) | 82 (82.8) |  |
| **Time to** **dialysis**- months | 20.4 ± 7.1 | 19 ± 7.1 | 0.190 |
| **Weigh**t - kg | 82.2 ± 25.1 | 80.7 ± 24 | 0.703 |
| **Body mass index** - kg/m^2^ | 29.4 ± 7.1 | 28.6 ± 6.7 | 0.459 |
| **Smoking status-**  no. (%) |  |  | 0.950 |
| Current | 13 (19.1) | 18 (18.2) |  |
| Former | 21 (30.9) | 29 (29.3) |  |
| Never | 34 (50) | 52 (52.5) |  |
| **Hba1c -** % | 6.9 ± 1.8 | 7.4 ± 2.1 | 0.156 |
| **Blood pressure** at baseline – mm Hg |  |  |  |
| - systolic blood pressure | 143.5 ± 16.1 | 139.4 ±18.4 | 0.143 |
| - diastolic blood pressure | 80.9 ± 9.6 | 78.3 ± 10.9 | 0.113 |
| **Blood pressure** at start of dialysis – mm Hg |  |  |  |
| - systolic blood pressure | 144.4 ± 19.2 | 145.7 ± 18.5 | 0.658 |
| - diastolic blood pressure | 77.3 ± 12.4 | 79.8 ±13.2 | 0.255 |
| **Estimated GFR** **at** baseline **-** ml/min/1.73m^2^ | 33.0 ± 8.7 | 35.8 ± 11 | 0.086 |
| **Estimated GFR** at start of dialysis– ml/min/1.73m^2^ | 14.5 ± 10.4 | 13.8 ± 7.3 | 0.632 |
| **Urinary albumin-to-creatinine ratio** at baseline – mg/g | 2749.6 ± 1464.2 | 2741.1 ± 1511.2 | 0.971 |
| **Urinary albumin-to-creatinine ratio** at start of dialysis – mg/g | 3260.8 ± 2463.5 | 4049.6 ± 2538.2 | 0.047 |

**Table S2.** **Characteristics of participants at start of dialysis according to treatment status.**

|  | **Dapagliflozin** |  | **Placebo** |  |
| --- | --- | --- | --- | --- |
|  | **Off-treatment**  **(n=40)** | **On-treatment**  **(n=28)** | **Off-treatment**  **(n=63)** | **On-treatment**  **(n=36)** |
| **Age** - yr | 57.3 ± 12.8 | 57.5 ± 15.2 | 56.4 ± 13.9 | 52.9 ± 15.3 |
| **Sex** (female) - no. (%) | 11 (27.5) | 6 (21.4) | 20 (31.7) | 11 (30.6) |
| **Race** – no. (%) |  |  |  |  |
| Asian | 10 (25.0) | 9 (32.1) | 16 (25.4) | 7 (19.4) |
| Black or African American | 2 (5.0) | 1 (3.6) | 0 (0) | 3 (8.3) |
| White | 20 (50.0) | 17 (60.7) | 36 (57.1) | 19 (52.8) |
| Other | 8 (20.0) | 1 (3.6) | 11 (17.5) | 7 (19.4) |
| **CV event before dialysis**- no. (%) |  |  |  |  |
| - No | 24 (60.0) | 16 (57.1) | 39 (61.9) | 22 (61.1) |
| - Yes | 16 (40.0) | 12 (42.9) | 24 (38.1) | 14 (38.9) |
| **Reason renal failure**- no. (%) |  |  |  |  |
| Acute renal failure | 12 (30.0) | 3 (10.7) | 10 (15.9) | 7 (19.4) |
| Progression of disease | 28 (70.0) | 25 (89.3) | 53 (84.1) | 29 (80.6) |
| **Planned/acute start**- no. (%) |  |  |  |  |
| Acute start | 26 (65.0) | 15 (53.6) | 27 (42.9) | 13 (36.1) |
| Planned start | 14 (35.0) | 13 (46.4) | 36 (57.1) | 23 (63.9) |
| **Time to** **dialysis**- months | 18.9 ± 6.8 | 22.7 ± 7.1 | 19.5 ± 7.1 | 18 ± 7.0 |
| **Weigh**t - kg | 78.4 ± 21.3 | 87.6 ± 29.3 | 81.4 ± 26.4 | 79.6 ± 19.4 |
| **Body mass index** kg/m^2^ | 28.5 ± 5.9 | 30.8 ± 8.5 | 29 ± 7.1 | 28 ± 6.2 |
| **Smoking status=** no.(%) |  |  |  |  |
| Current | 7 (17.5) | 6 (21.4) | 13 (20.6) | 5 (13.9) |
| Former | 13 (32.5) | 8 (28.6) | 16 (25.4) | 13 (36.1) |
| Never | 20 (50) | 14 (50) | 34 (54) | 18 (50) |
| **Hba1c** - % | 7.2 ± 2.0 | 6.5 ± 1.3 | 7.5 ± 2.1 | 7.0 ± 2.1 |
| **Blood pressure** at baseline – mm Hg |  |  |  |  |
| - systolic blood pressure | 142.2 ± 18.3 | 145.4 ± 12.4 | 138.5 ± 20 | 141.1 ± 15.4 |
| - diastolic blood pressure | 78.6 ± 9.4 | 84.1 ± 9.1 | 78.1 ± 10.8 | 78.5 ± 10.9 |
| **Blood pressure** at start of dialysis – mm Hg |  |  |  |  |
| - systolic blood pressure | 143.3 ± 19.1 | 145.9 ± 19.6 | 146.3 ± 18.5 | 144.6 ± 18.6 |
| - diastolic blood pressure | 77.2 ± 13.2 | 77.5 ± 11.4 | 79.7 ± 13.0 | 79.9 ± 13.8 |
| **Estimated GFR** at baseline- ml/min/1.73m^2^ | 33.6 ± 8.9 | 32.3 ± 8.5 | 36.9 ±12.3 | 34.0 ± 8.1 |
| **Estimated GFR** at start of dialysis- ml/min/1.73m^2^- | 17.6 ± 12.1 | 9.9 ± 4.6 | 14.9 ± 8.0 | 11.9 ± 5.5 |
| **Urinary albumin-to-creatinine ratio** at baseline | 2784.8 ± 1452.4 | 2699.2 ± 1506.3 | 2657.4 ± 1563.0 | 2887.6 ± 1425.5 |
| **Urinary albumin-to-creatinine ratio** at start of dialysis | 3470.9 ± 2656.5 | 2960.5 ± 2169.8 | 4129.8 ± 2754.3 | 3909.4 ± 2137.4 |

**Figure S1: Efficacy after initiation of dialysis according to treatment status**

**Alt text:** Forrest plot depicting all-cause mortality, CV-death and non-CV death for patients treated with dapagliflozin or placebo, who continued and discontinued treatment, showing no major differences on all-cause mortality between patients who continued or discontinued dapagliflozin after start of dialysis.

**Table S3: Safety after initiation of dialysis according to treatment status**

|  | **Dapagliflozin**  **(n=68)** |  | **Placebo**  **(n= 99)** |  |
| --- | --- | --- | --- | --- |
|  | **Continued treatment**  **(n=28)** | **Discontinued treatment**  **(n=40)** | **Continued treatment**  **(n=99)** | **Discontinued treatment**  **(n=63)** |
|  |  |  |  |  |
| Any adverse event - no. (%) | 9 (32) | 15 (38) | 11 (31) | 28 (44) |
| Any serious adverse event - no. (%) | 5 (18) | 10 (25) | 8 (22) | 19 (30) |
| Any adverse event, including death - no. (%) | 9 (32) | 12 (30) | 9 (25) | 23 (37) |
