# Supplementary figures and images for: Efficacy and safety of dapagliflozin in patients receiving dialysis: a post-hoc analysis of the DAPA-CKD trial
Source: Clin Kidney J. 2026 May 25;19(7):sfag170. doi: 10.1093/ckj/sfag170 (PMC13339949; doi:10.1093/ckj/sfag170)

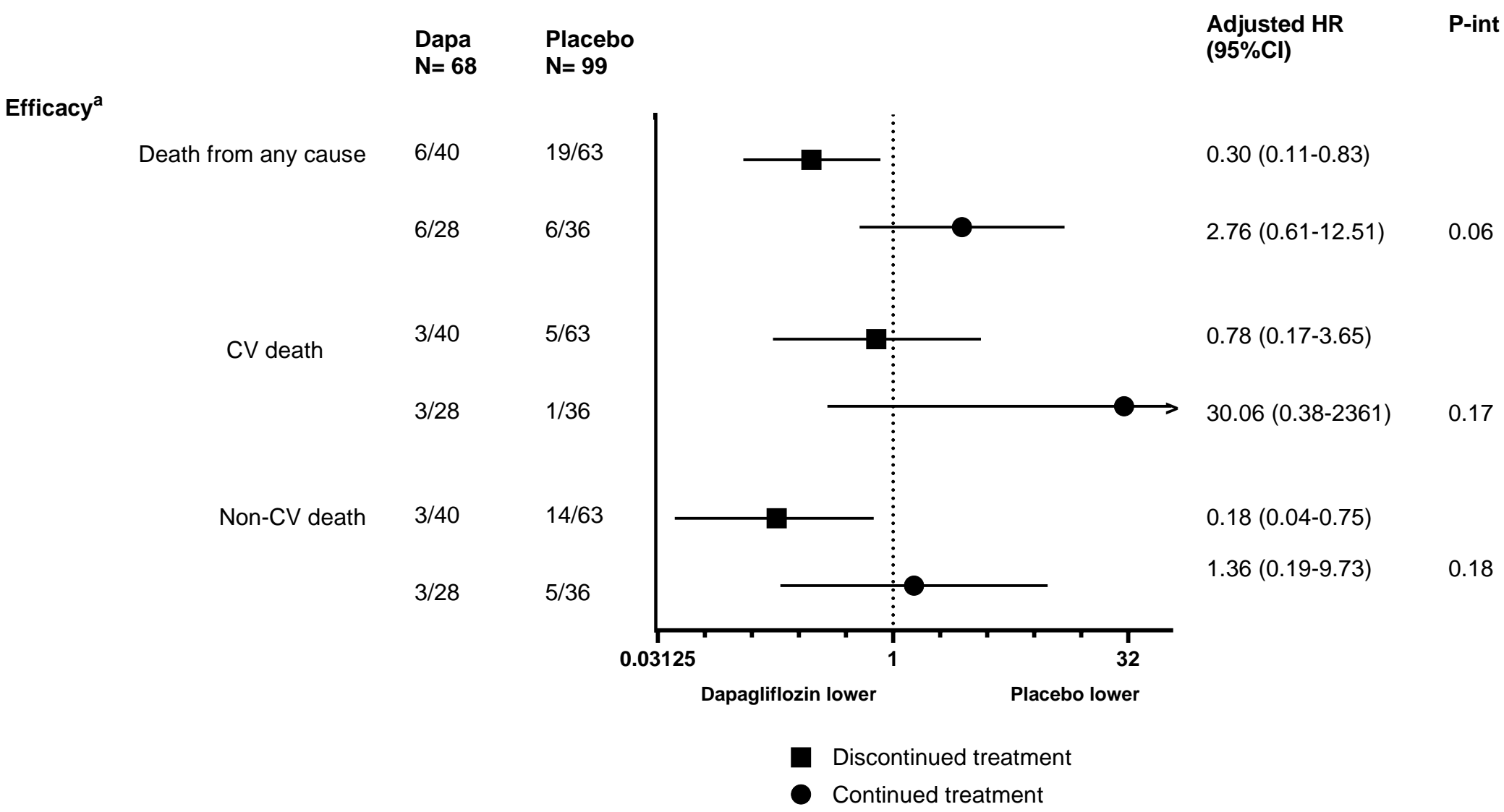

<sup>a</sup>Adjusted for: eGFR, UACR, age, sex and Hba1C using the last observed values before dialysis.

Supplement: sfag170_Supplemental_Files [file sfag170_supplemental_files.zip › Figure S1 Efficacy after initiation of dialysis according to treatment status.pdf]
